# Acute Infective Diarrhœa of Infants

**Published:** 1906-01-13

**Authors:** 


### ACUTE INFECTIVE DIARRHŒA OF INFANTS.

IN a recent clinical lecture Dr. Batten [1] deals with this disease on the basis of a special experience while in charge of a special ward set aside for the treatment of such cases at the hospital for sick children, Great Ormond Street.

In the first place, it is to be recognised that in the matter of nursing it is necessary to treat these patients as capable of infecting others by their discharges; to regard the disease as infective in the sense in which enteric fever is infective, and therefore as a disease to be treated with such precautions as enteric fever demands. With this object in view special nurses were appointed to remove soiled linen, and these nurses took no part in the feeding of the infants.

The characteristic features of acute infective diarrhœa in Dr. Batten's estimation are an acute onset attended by frequent vomiting; frequent motions of loose watery consistency, which in the early stage contain neither mucus nor blood, are green in colour and not offensive. The disease produces a condition of collapse; the extremities are cold and cyanosed, the eyes sunken, the skin shrivelled, and the abdomen generally lax and retracted. The pulse is feeble and small, and the temperature raised.

Out of 120 cases admitted for diarrhœa, 30 conformed to this type, and of these 18 died. The treatment advocated is as follows:—

1. Hypodermic injections of strychnia. A most valuable remedy for extreme collapse. Half a minim of the liquor strychninæ B.P. may be given and repeated in half an hour if necessary. Symptoms of poisoning may set in, but are not likely to be grave.

2. Infusion. Subcutaneous infusion of salt solution (one drachm to the pint of water). The infusion should be done by gravity; the needle should be passed well under the skin of the thorax, and the height of the column of water should be some 18 inches. The heat of the water in the glass receiver should be about 150° F. Four ounces should be infused, and the time occupied may be anything from 20 minutes to an hour.

3. The hot bath, with or without mustard (preferably with), is most valuable for conditions of collapse. The strength should be half an ounce of mustard to the gallon of water at a temperature of 100° F., rising to 110° F.

4. Washing out the stomach. This should be done by the mouth, for the nostril does not admit a tube large enough to allow the escape of curds of any size. A solution of sodium bicarbonate, two grains to the ounce, may be used for the lavage.

5. Rectal irrigation with hot water is performed by passing a rubber tube well into the rectum, and running in some six or eight ounces under a pressure of 12 inches; this to be repeated as often as is necessary.

6. Feeding is the most important matter. Milk in every form should be stopped. It is recommended that albumin water is as good as anything, in the proportion of the white of one egg to four ounces of water. Of this an ounce may be given every two hours, with 10 or 20 minims of brandy. This diet should be continued for three days at least. Then a little whey may be added, and gradually increased until whey and albumin water are being taken in equal quantity. At the end of a week a little citrated milk (one grain of sodium citrate to the ounce of milk) may be added. The essence of success is caution in the gradual expansion of the dietary.

7. Of stimulants, brandy is considered to be the best.

8. Of drugs, the most useful by far are calomel and castor oil.

[1] Clin. Jour., Jan. 3, 1906.

